# Predicting combinatorial binding of transcription factors to regulatory elements in the human genome by association rule mining

**DOI:** 10.1186/1471-2105-8-445

**Published:** 2007-11-15

**Authors:** Xochitl C Morgan, Shulin Ni, Daniel P Miranker, Vishwanath R Iyer

**Affiliations:** 1 Institute for Cellular and Molecular Biology and Center for Systems and Synthetic Biology, The University of Texas at Austin, Austin, Texas 78712-0159, USA; 2 Department of Computer Science, The University of Texas at Austin, Austin, Texas 78712-0159, USA

## Abstract

**Background:**

Cis-acting transcriptional regulatory elements in mammalian genomes typically contain specific combinations of binding sites for various transcription factors. Although some cis-regulatory elements have been well studied, the combinations of transcription factors that regulate normal expression levels for the vast majority of the 20,000 genes in the human genome are unknown. We hypothesized that it should be possible to discover transcription factor combinations that regulate gene expression in concert by identifying over-represented combinations of sequence motifs that occur together in the genome. In order to detect combinations of transcription factor binding motifs, we developed a data mining approach based on the use of association rules, which are typically used in market basket analysis. We scored each segment of the genome for the presence or absence of each of 83 transcription factor binding motifs, then used association rule mining algorithms to mine this dataset, thus identifying frequently occurring pairs of distinct motifs within a segment.

**Results:**

Support for most pairs of transcription factor binding motifs was highly correlated across different chromosomes although pair significance varied. Known true positive motif pairs showed higher association rule support, confidence, and significance than background. Our subsets of high-confidence, high-significance mined pairs of transcription factors showed enrichment for co-citation in PubMed abstracts relative to all pairs, and the predicted associations were often readily verifiable in the literature.

**Conclusion:**

Functional elements in the genome where transcription factors bind to regulate expression in a combinatorial manner are more likely to be predicted by identifying statistically and biologically significant combinations of transcription factor binding motifs than by simply scanning the genome for the occurrence of binding sites for a single transcription factor.

## Background

Substantial differences of phenotype can be primarily the result of differences in gene expression levels rather than in protein structure. Genes are dynamically regulated, primarily at the transcriptional level, by protein transcription factors that bind DNA at cis-regulatory regions to activate or repress expression. Mammalian cis-regulatory regions range in length from the 60 bp human muSK enhancer [1] to the 450 bp human TGFβ enhancer [2] to the 1100 bp enhancer of murine Pax6 [3], but they are generally a few hundred base pairs in length. Enhancers contain binding sites for transcription factors, sometimes for a single factor and sometimes for many [4]. A detailed understanding of the transcriptional regulatory programs of any organism requires knowledge of the binding sites of transcription factors, the circumstances and cellular conditions under which these transcription factors bind to their targets, and the genes that are regulated by combinations of transcription factors.

Cis-regulatory regions for most of the approximately 20,000 protein-coding genes encoded in the human genome have not yet been characterized [5]. Transcription factor binding sites, and thus cis-regulatory regions, can be identified using high-throughput methods such as ChIP-chip [6-9], but there are more than 2000 transcription factors encoded in the human genome [10,11]. This diversity of transcription factors, coupled with the fact that many are likely to be expressed and to combinatorially regulate target genes in a developmental, cell-, or tissue-specific manner, makes experimental identification of cis-regulatory regions challenging even with genome-wide ChIP-chip. Computational identification of cis-regulatory motifs based on signatures of their presence in the genomic sequence is an attractive alternative.

A major class of computational methods for identifying regulatory elements relies on the occurrence of TF binding sites in close proximity within regulatory elements. For example, the stripe 2 enhancer of the *even-skipped (eve) *gene in *Drosophila melanogaster *has twenty binding sites for four TFs within an area of roughly 600 bp [12]. The *knirps *gene of *Drosophila *is regulated by two enhancers containing six binding sites each for the transcription factors *bicoid *and *caudal *as well as two *hunchback *sites [13]. The HS2 enhancer of the human β-globin locus contains four NF-E1 binding sites and 2 CACC boxes within 250 bp [14], while a 300 bp region near the interleukin 2 transcriptional start site contains multiple binding sites for Ap-1 and Oct1 as well as sites for NFκB and NFAT [15]. Thus, the density of TF binding sites may be used as a means to locate cis-regulatory regions computationally [16].

Computational location of cis-regulatory modules by clustering of transcription factor binding sites has been implemented in genomes ranging from yeast [17] to human [18]. Previous approaches include "sliding window" [19-21] to Hidden Markov models [16,18] to position weight matrix clustering [22-26], while clusters have been defined both homotypically [19,21] and heterotypically [20,27-29]. These computational methods have been used to locate many cis-regulatory regions and novel target genes, notably in *Drosophila*. One limitation of these heterotypic clustering methods is the need to know which combinations of transcription factors should define the heterotypic clusters.

Numerous transcription factors are known to cooperate in certain contexts; for example, it is known that many genes involved in inflammation are regulated by Ap-1 and NFκB [30]. Similarly, interactions between PU.1 and GATA family TFs mediate cell differentiation in B-cell development [31]. Prediction of transcription factor cooperativity has been carried out in yeast [32,33] and human [34], but elucidation of the entire network of transcription factors that cooperate with one another in cis-regulatory regions is far from complete. In order to better define biologically relevant, heterogeneous combinations of transcription factors, we have developed an association rule data mining approach to search genome sequence information and identify over-represented adjacent motifs for transcription factor binding. Predicting transcription factor cooperation by data mining using association rules has previously been attempted in yeast as well as *C. elegans *and human chromosome 22 [35-37] but these attempts have been limited to mining known promoters [35] or repetitive elements such as microsatellites [36,37] rather than applied to the entire human genome.

Association rule data mining [38] was originally used in market basket analysis to determine which items are frequently purchased together. Basket analysis uses a database of transactions in which each tuple is a list of items purchased in one customer's transaction. Mining seeks to discover rules such as "spaghetti ⇒ parmesan cheese," meaning "People who buy spaghetti also often buy parmesan cheese." Association rules can be formally described as follows: [38]

• *I *= *{i*_1_, *i*_2_...*i*_*n*_*} *is a set of literals called items.

• *D *is a set of transactions. Each transaction *T *is a set of items such that *T *⊆ *I*.

• A transaction *T *contains *X*, a set of items in *I*, if *X *⊆ *T*.

• An association rule is an implication of *X *⇒ *Y*, where *X *⊂ *I*, *Y *⊂ *I*, and *X *∩ *Y *= ∅.

• *C *is the confidence of a rule *X *⇒ *Y *in transaction set *D *if *c*% of transactions in *D *that contain *X *also contain *Y*. It is also known as the conditional probability of Y given X, or P(Y|X).

• *S *is the support of rule *X *⇒ *Y *in set *D *if *s*% of transactions in *D *contain both X and Y. It is also known as the joint probability of both X and Y, or P(X ∩ Y).

If a rule *X *⇒ *Y *has high confidence, it is likely that transactions containing *X *will likely also contain *Y*. However, the existence of such a rule does not by itself imply any causal relationship between *X *and *Y*.

**Figure 1:**
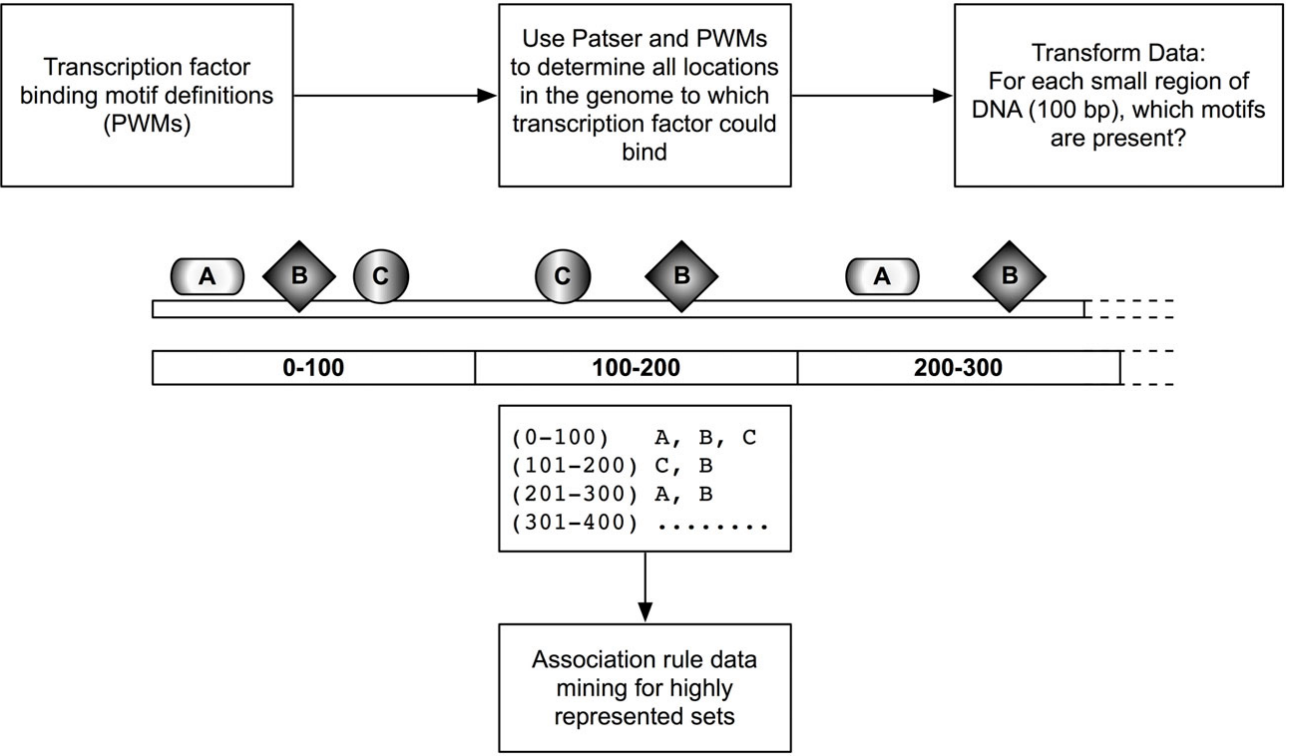
**Overview**. Patser is used to map all possible binding sites in the genome for each of 83 position weight matrices (PWMs) from TRANSFAC. The genome is then scored 100 bp at a time for the presence or absence of each PWM, and association rules are used to mine the genome for frequently co-occurring pairs.

Determining over-represented transcription factor partners may help to reveal biological roles for less well-studied transcription factors. Therefore, in our studies, we used data mining to determine whether two transcription factors whose experimentally determined binding motifs were frequently proximal to one another were also likely to have biologically meaningful interactions. For example, the rule "Nuclear Factor Kappa B ⇒ Ap-1" would indicate "Where there is a motif for NFκB, there is often also an Ap-1 motif." To allow application of association rules to transcription factor motifs in the human genome, we divided the genome into segments and scored each segment for the presence or absence of each of 83 transcription factor binding motifs (Figure 1). Thus, the set of 83 motifs becomes *I*, each individual transcription factor binding motif becomes an item, and each small segment of genome becomes a transaction *T *whose contents *X *are the motifs located within.

## Results

Our major aim was to determine whether a pair transcription factors whose motifs were frequently near one another were more likely to have a biological association than a pair of transcription factors whose motifs were not. In order to test this hypothesis, we located all possible binding sites in the human genome for the position weight matrices (PWMs) of each of 83 transcription factors (Additional file 1). We then divided the genome into 100 bp regions and used association rule data mining to calculate support and confidence for each transcription factor pair in the human genome.

**Figure 2:**
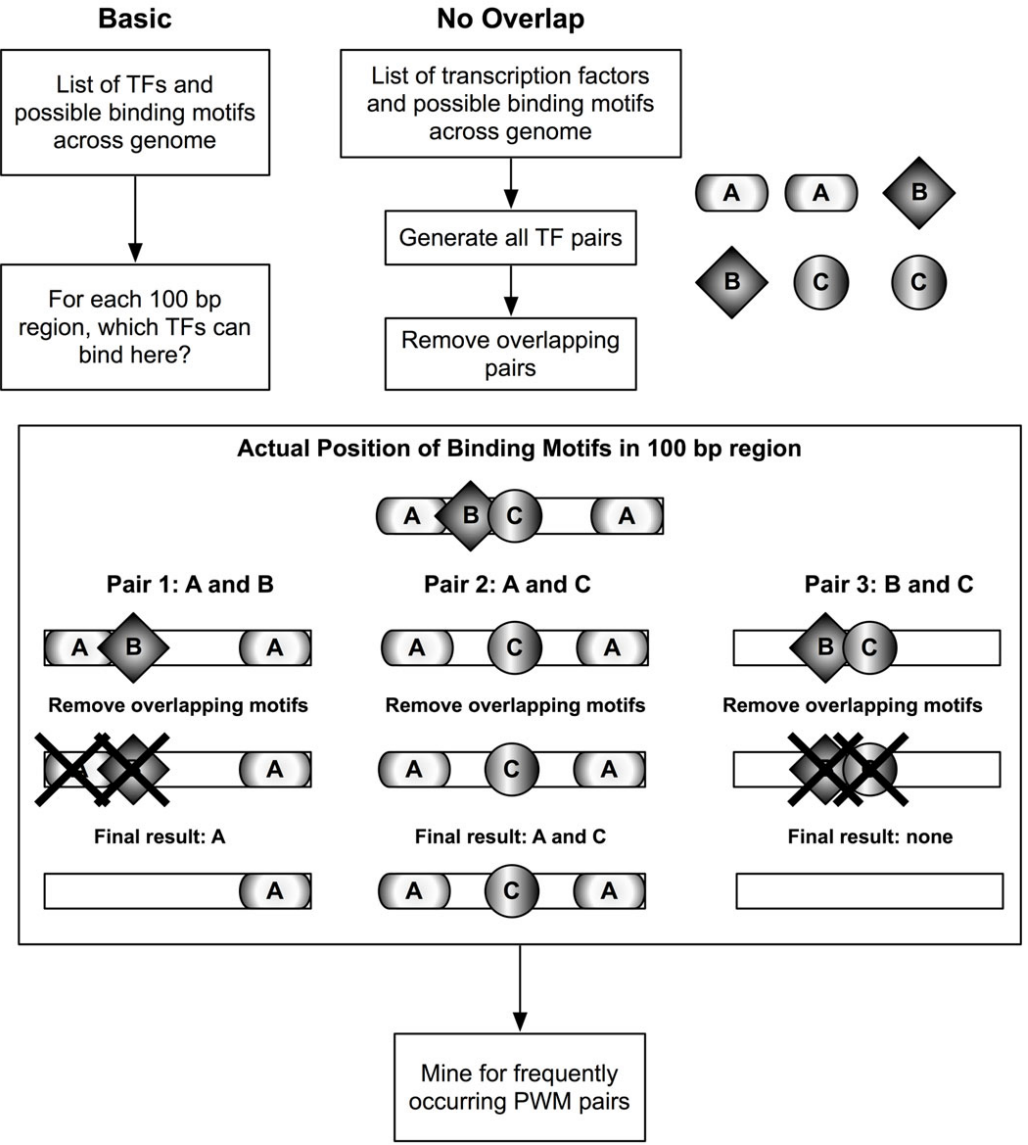
**Mining without overlap**. In order to avoid enriching primarily for TF pairs that bind similar motifs, the genome is mined once for each pair AB. All overlapping motifs between A and B are removed before calculating support, confidence, and *P*-values, then restored upon subsequent iterations.

Straightforward association rule mining that simultaneously considers all motif positions discovers high numbers of transcription factor pairs that bind identical or highly similar motifs. For example, two different transcription factors A and B may both bind to the motif "CACGTG", so the confidence *C *of the rule A ⇒ B will be 100%. Similarly, if A binds to "CACGTG" and B binds to "CACGTGA," this high overlap between binding motifs will result in the confidence being very high while the rule is neither interesting nor surprising, although it may still be biologically valid. To avoid discovery of enriched overlapping motifs, for each transcription pair AB, all overlapping binding sites between A and B were removed before calculating support and confidence (Figure 2). We also calculated a *P*-value based on the hypergeometric probability of observing the association between A and B by chance. We ensured that associations between transcription factor motifs were not an artifact caused by the presence of repetitive DNA, by considering repeat masked regions separately (Methods and Additional file 2). Furthermore, in order to rule out the possibility that associations were generated by nucleotide bias, we ascertained that dinucleotide and trinucleotide frequencies of segments containing motif pairs were not significantly different from segments containing one member of the pair or background (data not shown).

In order to determine whether biologically significant associations between PWMs arise in promoter regions, we applied the same pairwise mining algorithm to the subset of the genome that was 1 kb upstream of the transcriptional start site of all human RefSeq genes [39]. Because transcription factor function is often phylogenetically conserved, we also examined whether the combinations we identified by mining the human genome were identifiable in the mouse genome; we performed identical pairwise mining for significant associations among the same 83 transcription factors on mouse chromosome 1.

### Identifying meaningful TF pairs

**Figure 3:**
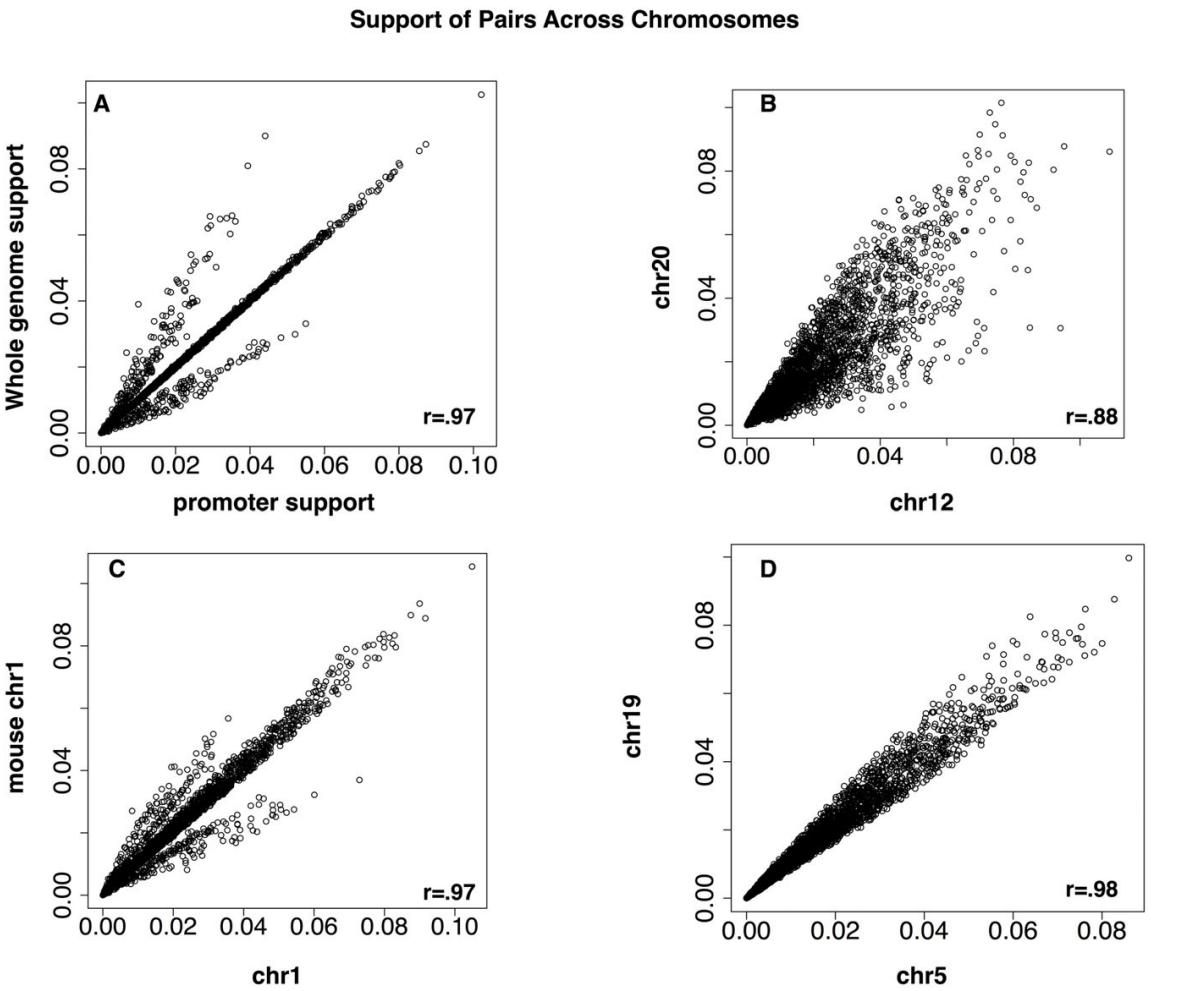
**Support of TF pairs across chromosomes**. The support of a given TF pair is highly correlated between chromosomes (B, D). This is also true for support in promoter regions versus the entire human genome (A) as well as support between human and mouse chromosomes (C).

Due to the size of the human genome and the tendency of PWMs to match at a large number of genomic locations, all TF pairs showed some co-occurrence. This support for possible transcription factor PWM pairs ranged from 9 x 10^-6 ^to 0.2. Support for the association of a given pair of transcription factors was highly conserved, not only between promoters and the entire genome (Figure 3A), but also between the human chromosomes and mouse chromosome 1 (Figure 3C) and between individual human chromosomes (Figure 3B, Figure 3D), suggesting that the associations revealed by mining are biologically relevant.

Association rules with the highest support and confidence are typically regarded as being interesting; however, if two different transcription factors each have large numbers of independent binding motifs in the genome, they could appear to be associated with high support values merely by chance. To minimize this possibility and to select those TF pairs occurring more frequently than by random chance and thus likely to be biologically meaningful, we also calculated the statistical significance (*P*-value) of observing each TF pair using the hypergeometric probability distribution. We defined the dataset "all" as the complete set of 3403 PWM pairs, and we selected three subsets with high confidence and significance for further analysis: "genomewide," "mouse," and "promoter" (Additional file 3).

The subsets "genomewide", "promoter", and "mouse" were defined as *P *< 0.05, greater than median difference between confidence A ⇒ B and confidence B ⇒ A. For the subset "genomewide" this was measured on the entire human genome and resulted in 66 TF pairs. For the subset "mouse," this was measured on mouse chromosome 1 and resulted in 184 pairs. For the subset "promoter", this was measured only across regions 1 kb upstream of the transcriptional start site of each RefSeq gene and resulted in 28 pairs.

The subsets of PWM pairs chosen for further inspection were of exceptionally high support and statistical significance. They co-occurred within the same short segment of DNA throughout the human genome much more often than the others, and much more frequently than expected by chance given their individual distributions. Transcription factors binding to the motifs represented by these PWMs were therefore expected to bind and jointly regulate the expression of target genes.

### Microarray verification

We hypothesized that high-support, high-significance TF pairs or their target genes might be co-expressed in microarray data more often than other pairs. Therefore, we calculated the Pearson correlations of expression for all genes across 4742 human microarrays from the Stanford Microarray Database, but we saw no difference between the expression correlations of selected TF pairs and all TF pairs and no difference between genes containing both members of a high-support, high-significance motif pair 1 kb upstream of the transcriptional start site and genes without (data not shown).

### Verification In the literature

**Figure 4:**
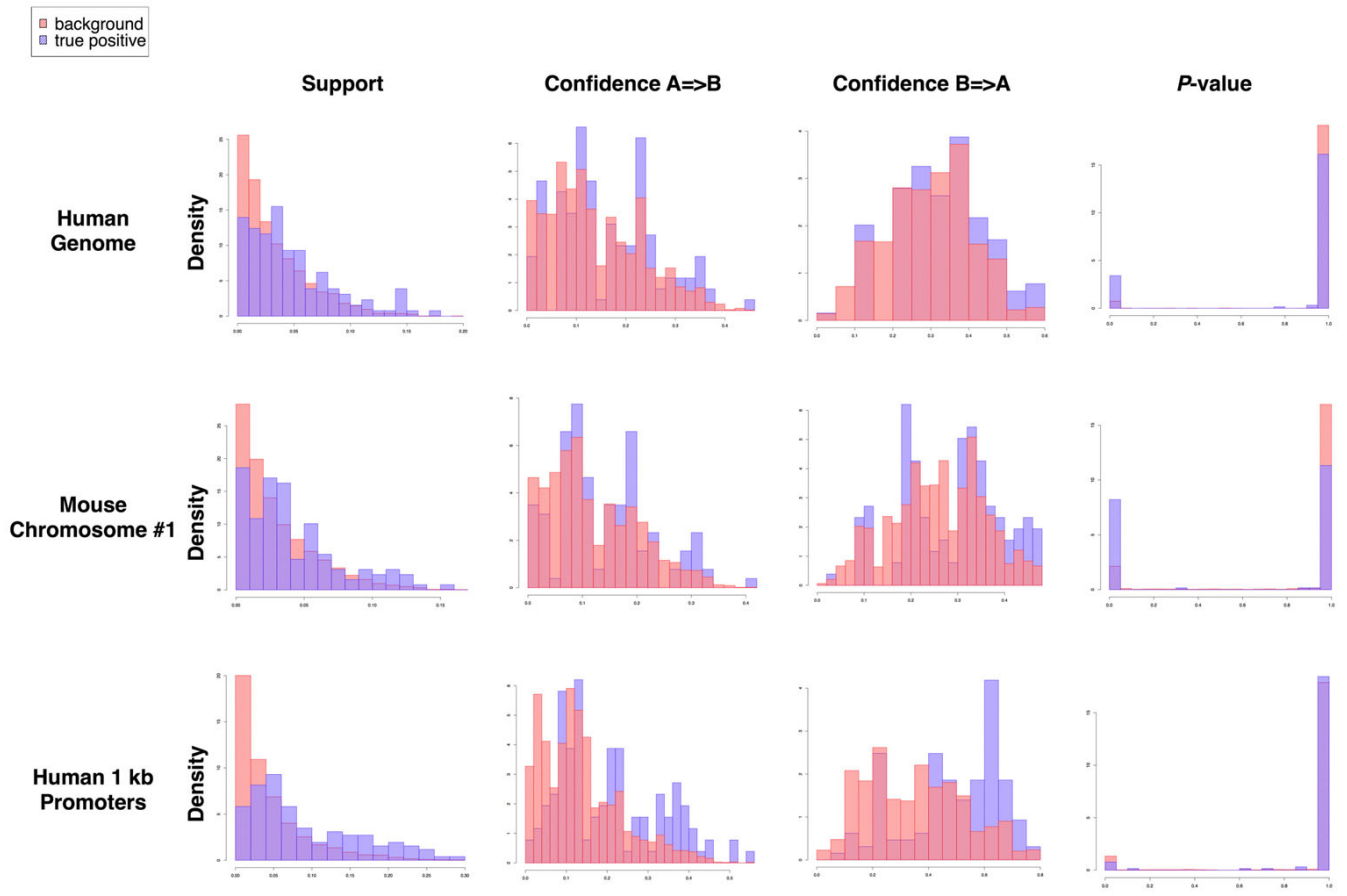
**Distributions of support, confidence, and *P*-value for true positives and all pairs**. Distribution histograms of support, confidence, and *P*-value for 131 true positives versus all pairs show higher support and confidence and lower *P*-values for true positives in the entire human genome, human promoter regions, and mouse chromosome 1.

We next manually examined the literature for evidence of biological associations and joint regulation of target genes by the "genomewide" and "mouse" subsets of PWM pairs that were identified by data mining. We found that many of these TF pairs were readily verifiable in the literature as true co-regulators of human and mouse genes (Table 1). For example the subsets "mouse" and "genomewide" both included the pair "Ap-2, Egr1." Genes known to be regulated by these two transcription factors include tumor necrosis factor α [40,41], human phenylethanolamine N-methyltransferase [42], and rat chromogranin B [43]. The subsets "mouse" and "genomewide" contain the pair "Sp1, p53"; each has been shown to regulate ICAM-1[44,45]. A comparison of distributions for all pairs compared to 131 true positives collected from the literature revealed that true positive pairs exhibited higher support and confidence and lower *P*-values than did all pairs (Figure 4), regardless of whether the entire human genome, human promoters, or mouse chromosome 1 were mined. As an exhaustive manual analysis of the literature for all TF pairs was not feasible, we used high-throughput co-citation analysis to further assess the biological relevance of the high-support, high-confidence TF pairs.

**Table 1:** High-confidence TF pairs verified in the literature.

**TF pair**	**Gene Regulated**	**Source**
Ap-2, p300	Mouse CITED4	[83]
Sp1, Gata2	Human PDGFβ receptor	[84]
Sp1, p300	Human ERK1	[126]
Ap-2, Egr1	Human tumor necrosis factor α, rat chromogranin B, human PNMT	[41–43, 67]
Ap-2, NFκB	Human tumor necrosis factor α	[41]
Egr1, Elk1	Human tumor necrosis factor α	[41, 132]
Egr1, Nf1	Human tissue factor pathway inhibitor 2	[40]
Egr1, p300	Human tumor necrosis factor α	[67]
Egr1, Sp1	Human TFPI-2, human SOD, human cd95, human TNFα	[40, 74, 97, 132]
Sp1, p53	Human Icam1	[44, 45]
Mzf1, Sp1	Human N-cadherin	[115]
Sp1, Srebp	Porcine LDL receptor, rat FAS	[123, 128]
Usf, Sp1	Rat FAS, human Top3, human liver fructose1,6 biphosphatase	[101, 119, 123]
Aml1, NFκB	Human GM-CSF	[66]
Aml1, Srebp	Human fatty acid synthase	[64]
Elk1, p300	Human tumor necrosis factor α	[132]
Gata2, NFκB	Human erythropoietin	[112]
Gata2, Sp1	Human PDGF receptor	[84]
Nf1, NFκB	Human tissue factor pathway inhibitor 2	[40]
NFκB, p300	Human I-gamma 1, mouse tapasin	[87, 111]
Pax5, p300	Human immunoglobin κ	[133]
Ap-1, NFκB	Human interleukin 6, human RANTES, human TNFα, human GM-CSF	[41, 60, 70, 77]

Examples of high-confidence TF pairs that could be verified in the literature as co-regulators of mammalian genes.

### High-throughput co-citation

In order to determine whether the members of a TF pair were co-cited in the literature more often than expected by chance and more often than the pairs that were not significant, we used the CoCiteStats package in R [46] to calculate PubMed co-citation rates for all TF pairs and subsets. For each pair of PWMs, CoCiteStats calculates co-citation by determining the concordance, Jaccard index, and Hubert's Γ, as well as the *P*-values for these indices, which are significant at *P *< 0.05 [47]. Concordance is a straightforward measure of how many papers in PubMed co-cite both genes. The Jaccard index is the ratio of the number of papers containing both genes to the number of papers containing at least one of the two genes. Hubert's Γ measures the degree of association between two binary variables, ranges from -1 to 1, and can be interpreted similarly to the Pearson correlation [47]. Because papers that cite a large number of genes are less likely to contain meaningful information about interactions between any two genes cited in that paper than papers citing fewer genes, CoCiteStats also weights data for paper size (number of genes cited in a paper), gene size (number of papers that cite a gene), and both gene and paper size [47].

**Figure 5:**
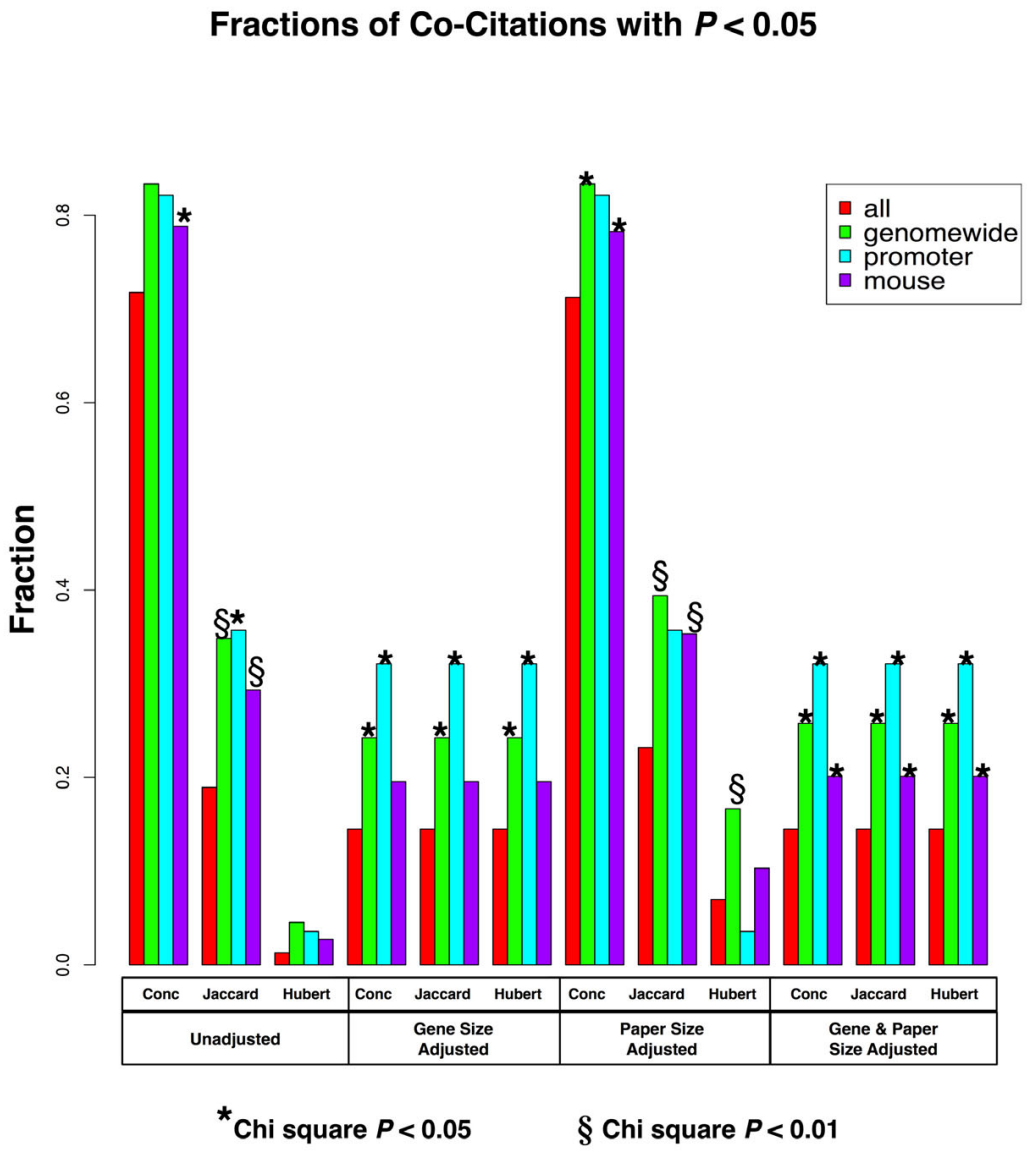
**Fractions of TF pairs with significant co-citation *P*-values**. Fractions of TF pairs with significant co-citation *P*-values (*P *< 0.05) in each dataset. Asterisks indicate a significant difference between all pairs and the selected subset as measured by a Chi square test. *P*-values significant after the Bonferroni correction for multiple hypothesis testing are indicated by "§".

Figure 5 shows the fraction of total TF pairs with significant co-citation *P*-values (*P *< 0.05) in each dataset. Asterisks indicate a significant difference between all TF pairs and the selected subset as measured by a Chi square test. All sets indicated by "§" were significant after Bonferroni correction for multiple hypothesis testing. All three subsets showed substantially higher proportions of TF pairs enriched for low co-citation *P*-values in all cases than the set of all pairs, indicating that transcription factors binding to the PWMs that showed substantial association with one another on the genome were more likely to be co-cited in the literature, reflecting a likely biological association between them. This enrichment of "genomewide" was significant for most values at all adjustments. The subset "mouse" was enriched for significant concordances and Jaccard values when unadjusted or adjusted by paper size and was significant for all values when adjusted by both gene and paper size. The subset "promoter" was more significant after adjustments for gene size or both gene and paper size.

## Discussion

Data mining using association rules discovered biologically meaningful cooperating TF pairs. Known true positive TF pairs showed higher support, confidence, and significance than did all pairs. Mined pairs with high significance as measured by the hypergeometric probability distribution and a large difference between confidence A=>B and confidence B=>A were frequently verified in the literature and showed enrichment of low co-citation *P*-values. We found that data mining the entire human genome was a better indicator of biological significance than was mining mouse chromosome 1, as measured by co-citation.

Given that phylogenetically conserved transcription factor binding motifs are thought to be biologically useful [48], it is interesting that 60% of the TF pairs in the subset "genomewide" were also present in the subset "mouse." Comparison of TF pairs for multiple mouse chromosomes or across more than two mammals may lead to even better results. The smaller overlap between "promoter" and "mouse" (14%) and "promoter" and "genomewide" (42%) may be due in part to differences in sequence size and nucleotide frequency; the sequence mined for "promoter" was a tenth the size of the sequence mined for "mouse" and ~1/200 the size of the sequence for "genomewide." Furthermore, the "promoter" sequence has a much higher GC content of 53% GC, while the human genome and mouse chromosome 1 are 41% GC; the PWMs used for mining have an average GC content of 46%.

Approximately 2900 of the TF pairs in our analysis were non-significant on mouse chromosome 1, human 1 kb promoter regions, or the human genome. The most confident of the remaining ~550 TF pairs may merit further study. Our estimated error rates for PWM matches located by Patser ranged from 3.5% to 61.5% with an average of 20% and a median of 18% (Additional file 4). Pairs containing a TF with a very high error rate are less likely to be of predictive value, but most TF pairs with high confidence and significance did not have very high Patser error rates.

Our approach is novel, low-cost, and straightforward to implement. The main advantage of this approach is that the signal for the association of transcription factors is detectable using only the genome sequence and is not limited by lack of prior knowledge about physiological conditions or cell types in which the transcription factor combination may be active. Unlike clustering algorithms, which require items to be assigned to only one cluster, association rules allow items to be members of many groups and may discover these relationships. This algorithm also enables us to analyze a great number of motifs and large amount of sequence data for which Gibbs sampling is not currently feasible. One limitation of our current implementation is that we have applied it to identify only combinations of two distinct transcription factors. Although it is possible to discover associations of multiple transcription factors in the genome sequence through association rule mining, this is more computationally demanding.

As with any computational prediction, the significant challenge is verification of the predicted TF pairs. Co-citation analysis was particularly useful given that expected measures of biological association between the members of predicted TF pairs, such as correlated expression of target genes and network connectivity, were not useful. There are several possible explanations for why we did not observe correlations for significant mined TF pairs in microarray data. First, the activity of transcription factors may not be primarily regulated transcriptionally. Rather, transcription factors may require degradation of chaperones to become active, as does NFκB, or ligand binding may be needed to cause an active receptor to relocalize to the nucleus, as in the case of the estrogen receptor. While some transcription factors, such as targets of immediate early genes, may have similar mechanisms of transcriptional activation, it is likely that many, if not most cooperating transcription factors will have diverse means of transcriptional regulation and will thus not be co-expressed. Furthermore, due to noise and the fact that transcription factors may be inactive in many cell types and experimental conditions, any co-expression signature may be lost in large amounts of microarray data even for transcription factors known to be co-expressed. For example, across the 4247 microarrays we analyzed, the Pearson correlations for Fos with JunB and Jun were -0.11 and 0.146, respectively; the correlation was -0.116 for Gata2 and Gata3 and 0.24 for Sox5 and Sox6. Thus, even for known pairs of transcription factors, there is little detectable coexpression across a large microarray dataset.

We found that genes containing significant pairs of PWMs in their promoters were no more likely to be co-regulated than a background set. One possible explanation is that our list of 4742 microarrays represented a wide variety of experimental conditions, but many of the transcription factors we studied are active only under specific conditions satisfied in only a small number of experiments. Furthermore, the short, degenerate nature of position weight matrices means that thousands of 1 kb upstream regions are likely to contain any given PWM pair. We found that each PWM was present in the upstream regions of 10,948 genes on average, while the promoter region of each gene contained an average of 70 PWMs (data not shown). Thus, any comparisons of subsets became comparisons of most genes versus most genes, making it difficult to detect a change in the distribution of correlation coefficients. Observing correlated expression of the target genes of highly supported TF pairs would be much more likely if target genes could be more rigidly defined and a subset of microarray experiments was chosen to reflect likely conditions for transcription factor activity, but choosing these experiments is nontrivial, particularly for transcription factors that have not been well-studied.

Co-citation is not without drawbacks. The fact that two proteins are cited in a paper does not necessarily mean that they interact with one another. Furthermore, well-studied proteins are likely to be overrepresented while less-studied proteins will be missed. Validation by co-expression, however, requires knowledge of target genes and conditions for transcription factor activity; this may not be known or be feasible for experimental analysis. Future experimental validation of predicted associations could be accomplished by identifying binding targets for these transcription factors by genome-wide chromatin immunoprecipitation analyses and determining joint occupancy of target promoters by predicted combinations of transcription factors. Current maps of human protein-protein interactions [49-53] may not yet define many interactions for human transcription factors or may contain high rates of false positives [54], but they are constantly improving. We anticipate that better human protein-protein interaction maps will eventually provide a superior means of assessing performance of TF pair data mining, allowing this method to be refined to reveal both novel transcription factor interactions and biological context for previously uncharacterized transcription factors.

## Conclusion

Here we have described a novel genomic method for predicting biologically relevant, heterogeneous combinations of cooperating transcription factors by data mining using association rules to search genome information and identify over-represented proximal motifs. Using this approach, we show that that true positive cooperating TF pairs tend to have higher support, confidence, and significance, and that mined TF pairs with high confidence and significance are frequently verified in the literature and enriched for low co-citation *P*-values. Data mining the entire human genome enabled better discovery of biologically meaningful pairs than mining mouse chromosome 1, as measured by co-citation.

## Methods

### Data transformation

We collected 163 human position weight matrices (PWMs) from TRANSFAC [55] and removed those which were redundant or could not be mapped to RefSeq genes [39], leaving 83 PWMs for analysis (Additional file 1). We used Patser [56] to map all locations in the human genome assembly hg17 and in the repeat-masked human genome assembly hg18 [57] to which each transcription factor could bind with *P *< 0.001. We then divided the genome into 100 bp regions and scored each region for the presence or absence of each PWM. We chose a region size of 100 bp because it is compatible with the size of known cis-regulatory regions and large enough to contain multiple non-overlapping transcription factor binding motifs. PWMs tend towards large numbers of possible binding sites in the genome; 100 bp regions are small enough to prevent most regions from containing most motifs. We mined this matrix of genomic regions and motifs they contained for frequent itemsets, using association rules to search for *X *⇒ *Y *with high support *S*. Support and confidence were highly correlated between hg17 without repeat masking and hg18 with repeat masking (Additional file 2). High-support, high-confidence, significant PWM pairs were comparable between region sizes ranging from 75 bp to 225 bp, although larger region sizes yielded greater numbers of significant pairs.

### Estimating Patser error rate for PWMs

We estimated Patser error rates for each position weight matrix by calculating its average *P*-value across the genome as given by Patser, multiplying this by the size of the genome minus the length of masked repeats and then dividing by total number of matches to approximate the number of overestimated Patser matches.

### Mining without overlap

In order to avoid enrichment of PWMs with highly similar binding motifs, we mined the human genome without allowing motif overlap, one motif pair at a time. That is, for each TF pair AB (83 transcription factors taken two at a time, or 3403 pairs), after all possible binding motifs for A and B respectively were identified, any overlapping A and B motifs were removed before assigning the remaining non-overlapping sites to their respective 100 bp regions (Figure 2). The full set of matches for each factor was restored at the beginning of each iteration, so overlaps between A and C were unaffected by overlaps between A and B. For example, if transcription factor A had a binding motif of width 5 which was present at positions 100, 130, and 150, while factor B had a binding motif of width 7 present at 102, 160, and 175, the binding sites 100A and 102B would be removed from calculations due to overlap; the remaining binding sites would still allow the region from 100 to 200 to be scored as containing A and B. After scoring each 100 bp region, we calculated association rule support (proportions of regions) for A, B, and AB for each pair on each chromosome, correcting for the proportion of the genome that was repeat-masked. Additionally, we calculated confidence for A ⇒ B and B ⇒ A and a *P*-value based on the hypergeometric probability of observing the association between A and B by chance, given the individual distributions of their binding motifs in the genome, again correcting for repeat masking. To allow phylogenetic comparison and comparison of promoters versus the entire genome, we performed identical pairwise mining on mouse chromosome 1 and on the subset of the human genome that was 1 kb upstream from the transcriptional start site of all human RefSeq genes.

### Microarray data

To determine whether transcription factor pairs with high support and high confidence were highly co-expressed, we downloaded and analyzed a dataset consisting of 4742 human microarrays from the Stanford Microarray Database [58] and calculated the Pearson correlation for each gene pair with 100 or more experimental data points. We defined a list of potential target genes for TF pairs by scanning 1 kb upstream from the transcriptional start site of each RefSeq gene for each PWM.

### True positives

From the Compel database [59] and the literature, we collected 131 transcription factor pairs known to co-regulate mammalian genes [40,41,44,45,60-131].

## Software availability and requirements

Project name: Miner

Project home page: 

Operating system(s): All POSIX (Linux/BSD/UNIX-like) operating systems.

Programming language: C++

License: Academic Free License

## Authors' contributions

XM carried out data analysis. XM and SN wrote the computer code. XM and VI wrote the manuscript. DM provided the initial impetus for the design of this project. All authors participated in the design of the study. All authors read and approved the final manuscript.

## Supplementary Material

Additional file 1 **83 transcription factors from TRANSFAC**. A list of the 83 transcription factor position weight matrices from TRANSFAC used for this analysis. Click here for file

Additional file 2 **Effects of repeat masking**. Support, confidence A=>B, and confidence B=>A are highly correlated between hg17 without repeat masking and hg18 with repeat masking. Click here for file

Additional file 3 **The subsets "genomewide", "mouse", and "promoter"**. "Genomewide", "Promoter", and "Mouse" are defined as top 50% difference between confidence A=>B and confidence B=>A and *P *< 0.05 as measured by the hypergeometric distribution. Pairs indicated in bold have been verified in the literature. Click here for file

Additional file 4 **Estimated Patser error rates for PWMs**. Approximate overestimation rates of position weight matrices from Patser. Click here for file
