# Supplementary material for: Predicting combinatorial binding of transcription factors to regulatory elements in the human genome by association rule mining
Source: BMC Bioinformatics. 2007 Nov 15;8:445. doi: 10.1186/1471-2105-8-445 (PMC2211755; doi:10.1186/1471-2105-8-445)
Supplement: Additional file 1 — 83 transcription factors from TRANSFAC. A list of the 83 transcription factor position weight matrices from TRANSFAC used for this analysis. [file 1471-2105-8-445-S1.doc]

**Additional Table 1 – 83 transcription factors from TRANSFAC**

AML1_01

AP1_Q2

AP2_Q6

AP4_Q6

AREB6_03

ARNT_01

ARP1_01

ATF_01

BRN2_01

CART1_01

CDP_02

CEBP_C

CHOP_01

COUP_01

CREB_01

CREBP1_01

CREL_01

E2F_01

E2F_02

E47_02

EGR1_01

ELF1_01

ELK1_02

ER_Q6

FOXD3_01

FOXJ2_02

FREAC2_01

FREAC3_01

FREAC4_01

FREAC7_01

GATA2_01

GATA3_01

GATA_C

GRE_C

HFH3_01

HLF_01

HNF1_01

HNF4_01

HSF1_01

HSF2_01

IRF1_01

ISRE_01

LMO2COM_02

MAX_01

MEF2_03

MEF2_04

MEIS1_01

MIF1_01

MYB_Q6

MYCMAX_01

MYOD_Q6

MZF1_01

NF1_Q6

NFAT_Q6

NFE2_01

NFKAPPAB_01

NFY_01

NKX61_01

NRSF_01

OCT_C

P300_01

P53_01

PAX2_01

PAX5_01

PAX6_01

PBX1_02

RFX1_01

RORA1_01

RORA2_01

RREB1_01

SOX9_B1

SP1_Q6

SREBP1_02

SRF_Q6

SRY_02

STAT_01

TATA_01

TCF11_01

TGIF_01

TST1_01

USF_01

XBP1_01

YY1_02

A list of the 83 transcription factor position weight matrices from TRANSFAC used for this analysis.
