# Supplementary figures and images for: Predicting combinatorial binding of transcription factors to regulatory elements in the human genome by association rule mining
Source: BMC Bioinformatics. 2007 Nov 15;8:445. doi: 10.1186/1471-2105-8-445 (PMC2211755; doi:10.1186/1471-2105-8-445)

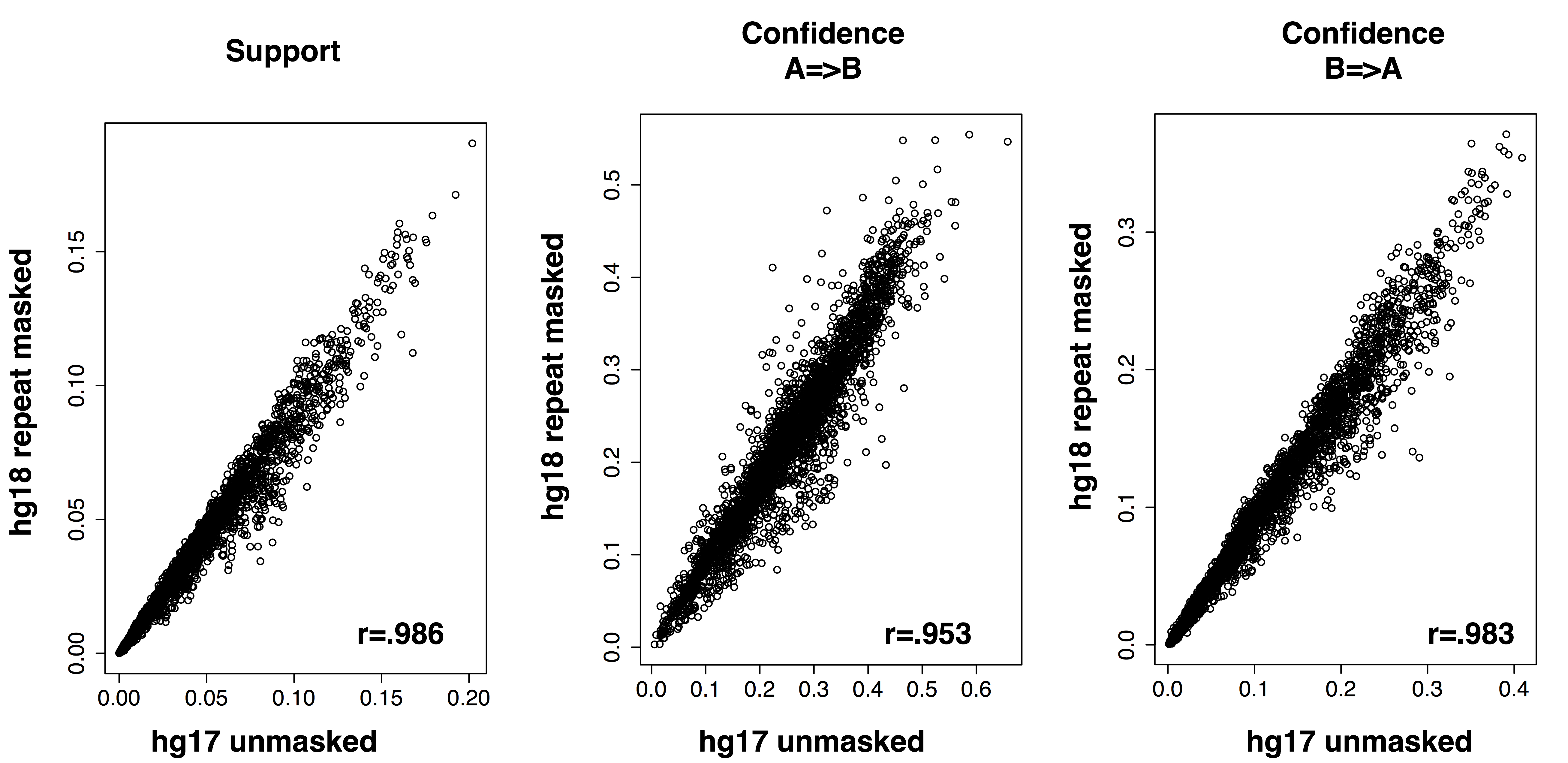

Supplement: Additional file 2 — Effects of repeat masking. Support, confidence A=>B, and confidence B=>A are highly correlated between hg17 without repeat masking and hg18 with repeat masking. [file 1471-2105-8-445-S2.tiff]
