# Supplementary material for: Predicting combinatorial binding of transcription factors to regulatory elements in the human genome by association rule mining
Source: BMC Bioinformatics. 2007 Nov 15;8:445. doi: 10.1186/1471-2105-8-445 (PMC2211755; doi:10.1186/1471-2105-8-445)
Supplement: Additional file 3 — The subsets "genomewide", "mouse", and "promoter". "Genomewide", "Promoter", and "Mouse" are defined as top 50% difference between confidence A=>B and confidence B=>A and P < 0.05 as measured by the hypergeometric distribution. Pairs indicated in bold have been verified in the literature. [file 1471-2105-8-445-S3.doc]

**Additional file 3 - the subsets “genomewide”, “mouse”, and “promoter”**

**Genomewide**

AML1_01.EGR1_01

AML1_01.MYCMAX_01

AP2_Q6.ARNT_01

AP2_Q6.ATF_01

AP2_Q6.E47_02

**AP2_Q6.EGR1_01**

AP2_Q6.NRSF_01

AP2_Q6.MAX_01

AP2_Q6.MYCMAX_01

AP2_Q6.MZF1_01

**AP2_Q6.NFKAPPAB_01**

AP2_Q6.P53_01

AP2_Q6.SREBP1_02

AREB6_03.ARNT_01

AREB6_03.EGR1_01

AREB6_03.NRSF_01

AREB6_03.P53_01

AREB6_03.SREBP1_02

ARNT_01.ER_Q6

ARNT_01.ELK1_02

ARNT_01.MYOD_Q6

ARNT_01.MZF1_01

ARNT_01.P300_01

ARNT_01.PAX2_01

ARNT_01.PAX5_01

ARNT_01.RREB1_01

ARNT_01.SP1_Q6

ARP1_01.EGR1_01

ATF_01.SP1_Q6

E47_02.MZF1_01

**EGR1_01.ELK1_02**

EGR1_01.ER_Q6

EGR1_01.HNF4_01

EGR1_01.MYOD_Q6

EGR1_01.MZF1_01

**EGR1_01.NF1_Q6**

**EGR1_01.P300_01**

EGR1_01.PAX2_01

EGR1_01.PAX5_01

EGR1_01.RREB1_01

**EGR1_01.SP1_Q6**

EGR1_01.YY1_02

ELK1_02.NRSF_01

ER_Q6.NRSF_01

MAX_01.MYOD_Q6

MAX_01.MZF1_01

**MAX_01.SP1_Q6**

MYCMAX_01.MZF1_01

MYCMAX_01.PAX5_01

**MYCMAX_01.SP1_Q6**

MYOD_Q6.NFKAPPAB_01

MYOD_Q6.NRSF_01

MYOD_Q6.SREBP1_02

MZF1_01.NRSF_01

MZF1_01.P53_01

**MZF1_01.SP1_Q6**

NF1_Q6.NRSF_01

NRSF_01.P300_01

NRSF_01.RREB1_01

NRSF_01.SP1_Q6

NRSF_01.PAX2_01

NRSF_01.PAX5_01

NRSF_01.YY1_02

**P53_01.SP1_Q6**

**SP1_Q6.SREBP1_02**

**SP1_Q6.USF_01**

**Promoter**

AREB6_03.EGR1_01

AREB6_03.NRSF_01

CART1_01.FOXD3_01

CART1_01.SRY_02

CDP_02.FOXD3_01

CDP_02.GATA_C

CDP_02.SOX9_B1

CDP_02.SRY_02

CREBP1_01.FOXD3_01

CREBP1_01.SRY_02

E2F_02.MZF1_01

E2F_02.NRSF_01

**EGR1_01.ELK1_02**

EGR1_01.MZF1_01

**EGR1_01.P300_01**

EGR1_01.PAX2_01

EGR1_01.PAX5_01

EGR1_01.YY1_02

FOXD3_01.MEF2_03

FREAC3_01.SRY_02

FREAC4_01.GATA_C

FREAC4_01.SRY_02

HNF1_01.TST1_01

IRF1_01.SRY_02

MEF2_03.SRY_02

MZF1_01.NRSF_01

PBX1_02.SRY_02

**Mouse**

AML1_01.ARNT_01

AML1_01.ATF_01

AML1_01.E2F_02

AML1_01.EGR1_01

AML1_01.MYCMAX_01

**AML1_01.NFKAPPAB_01**

AML1_01.NRSF_01

AML1_01.P300_01

**AML1_01.SP1_Q6**

**AML1_01.SREBP1_02**

AML1_01.USF_01

AML1_01.YY1_02

**AP1_Q2.NFKAPPAB_01**

AP2_Q6.ARNT_01

AP2_Q6.ATF_01

AP2_Q6.E2F_02

AP2_Q6.E47_02

**AP2_Q6.EGR1_01**

**AP2_Q6.GATA2_01**

AP2_Q6.MAX_01

AP2_Q6.MYCMAX_01

AP2_Q6.MZF1_01

**AP2_Q6.NFKAPPAB_01**

AP2_Q6.NRSF_01

**AP2_Q6.P300_01**

AP2_Q6.P53_01

AP2_Q6.SREBP1_02

AP2_Q6.USF_01

AREB6_03.ARNT_01

AREB6_03.ATF_01

AREB6_03.E2F_02

AREB6_03.EGR1_01

AREB6_03.GATA2_01

AREB6_03.MYCMAX_01

AREB6_03.MYOD_Q6

AREB6_03.MZF1_01

AREB6_03.NF1_Q6

AREB6_03.NRSF_01

AREB6_03.P300_01

AREB6_03.SREBP1_02

AREB6_03.USF_01

ARNT_01.ELK1_02

ARNT_01.MYOD_Q6

ARNT_01.MZF1_01

ARNT_01.P300_01

ARNT_01.PAX5_01

ARNT_01.SP1_Q6

ARNT_01.YY1_02

ARP1_01.ATF_01

ARP1_01.EGR1_01

ARP1_01.MYOD_Q6

ARP1_01.MZF1_01

ARP1_01.NRSF_01

ARP1_01.SREBP1_02

ATF_01.CREL_01

ATF_01.ELK1_02

ATF_01.ER_Q6

ATF_01.HNF4_01

ATF_01.MEIS1_01

ATF_01.MYB_Q6

ATF_01.MYOD_Q6

ATF_01.MZF1_01

ATF_01.NF1_Q6

ATF_01.NFE2_01

ATF_01.P300_01

ATF_01.PAX2_01

CDP_02.FOXD3_01

CDP_02.FOXJ2_02

CDP_02.NKX61_01

CREBP1_01.FOXD3_01

CREBP1_01.FOXJ2_02

CREBP1_01.NKX61_01

CREL_01.E2F_02

CREL_01.EGR1_01

CREL_01.MZF1_01

CREL_01.NRSF_01

CREL_01.SREBP1_02

E2F_02.ELK1_02

E2F_02.ER_Q6

E2F_02.GATA2_01

E2F_02.MYB_Q6

E2F_02.MYOD_Q6

E2F_02.MZF1_01

E2F_02.NF1_Q6

E2F_02.P300_01

E47_02.MZF1_01

E47_02.P300_01

**EGR1_01.ELK1_02**

EGR1_01.ER_Q6

EGR1_01.GATA2_01

EGR1_01.HNF4_01

EGR1_01.MYOD_Q6

EGR1_01.MZF1_01

**EGR1_01.NF1_Q6**

**EGR1_01.P300_01**

EGR1_01.PAX2_01

EGR1_01.PAX5_01

EGR1_01.RREB1_01

**EGR1_01.SP1_Q6**

EGR1_01.YY1_02

ELK1_02.MYCMAX_01

ELK1_02.MZF1_01

**ELK1_02.NFKAPPAB_01**

ELK1_02.NRSF_01

**ELK1_02.P300_01**

ELK1_02.SREBP1_02

ER_Q6.MYCMAX_01

ER_Q6.MZF1_01

ER_Q6.NFKAPPAB_01

ER_Q6.NRSF_01

FOXD3_01.HNF1_01

FOXD3_01.NKX61_01

FOXJ2_02.HLF_01

FOXJ2_02.HNF1_01

GATA2_01.MYCMAX_01

**GATA2_01.NFKAPPAB_01**

GATA2_01.NRSF_01

**GATA2_01.SP1_Q6**

GATA2_01.SREBP1_02

GATA2_01.USF_01

HNF4_01.NFKAPPAB_01

HNF4_01.NRSF_01

HSF2_01.MZF1_01

MAX_01.MYOD_Q6

MAX_01.MZF1_01

MAX_01.P300_01

**MAX_01.SP1_Q6**

MEF2_03.NKX61_01

MEF2_04.NKX61_01

MEIS1_01.NFKAPPAB_01

MEIS1_01.NRSF_01

MEIS1_01.SP1_Q6

MEIS1_01.SREBP1_02

MYB_Q6.NFKAPPAB_01

MYB_Q6.NRSF_01

MYB_Q6.SP1_Q6

MYB_Q6.SREBP1_02

MYCMAX_01.MYOD_Q6

MYCMAX_01.MZF1_01

MYCMAX_01.NF1_Q6

MYCMAX_01.NFE2_01

MYCMAX_01.P300_01

MYCMAX_01.PAX2_01

MYCMAX_01.PAX5_01

**MYCMAX_01.SP1_Q6**

MYCMAX_01.YY1_02

MYOD_Q6.NFE2_01

MYOD_Q6.NFKAPPAB_01

MYOD_Q6.NRSF_01

MYOD_Q6.PAX5_01

**MYOD_Q6.SP1_Q6**

MYOD_Q6.SREBP1_02

MYOD_Q6.USF_01

MYOD_Q6.YY1_02

MZF1_01.NFE2_01

MZF1_01.NFKAPPAB_01

MZF1_01.NRSF_01

MZF1_01.P53_01

MZF1_01.PAX2_01

MZF1_01.PAX5_01

**MZF1_01.SP1_Q6**

MZF1_01.SREBP1_02

MZF1_01.USF_01

MZF1_01.YY1_02

**NF1_Q6.NFKAPPAB_01**

NF1_Q6.NRSF_01

**NF1_Q6.SP1_Q6**

NF1_Q6.SREBP1_02

**NFKAPPAB_01.P300_01**

NKX61_01.SRY_02

NRSF_01.P300_01

NRSF_01.PAX2_01

NRSF_01.PAX5_01

NRSF_01.RREB1_01

NRSF_01.SP1_Q6

NRSF_01.YY1_02

**P300_01.PAX5_01**

**P300_01.SP1_Q6**

P300_01.SREBP1_02

P300_01.USF_01

**P300_01.YY1_02**

**P53_01.SP1_Q6**

**SP1_Q6.USF_01**

**The subsets “genomewide”, “mouse”, and “promoter” are defined as top 50% difference between confidence A=>B and confidence B=>A and *P* < 0.05 as measured by the hypergeometric distribution. Pairs indicated in bold have been verified in the literature.**
