# Supplementary material for: Predicting combinatorial binding of transcription factors to regulatory elements in the human genome by association rule mining
Source: BMC Bioinformatics. 2007 Nov 15;8:445. doi: 10.1186/1471-2105-8-445 (PMC2211755; doi:10.1186/1471-2105-8-445)
Supplement: Additional file 4 — Estimated Patser error rates for PWMs. Approximate overestimation rates of position weight matrices from Patser. [file 1471-2105-8-445-S4.doc]

**Additional file 4 – estimated Patser error rate for PWMs**

| **TF** | **Est. Error** |
| --- | --- |
| AML1_01 | 0.19 |
| AP1_Q2 | 0.18 |
| AP2_Q6 | 0.07 |
| AREB6_03 | 0.10 |
| ARNT_01 | 0.30 |
| ARP1_01 | 0.11 |
| ATF_01 | 0.41 |
| BRN2_01 | 0.21 |
| CART1_01 | 0.26 |
| CDP_02 | 0.54 |
| CEBP_C | 0.20 |
| CHOP_01 | 0.21 |
| COUP_01 | 0.12 |
| CREBP1_01 | 0.61 |
| CREB_01 | 0.51 |
| CREL_01 | 0.15 |
| E2F_01 | 0.44 |
| E2F_02 | 0.52 |
| E47_02 | 0.09 |
| EGR1_01 | 0.17 |
| ELF1_01 | 0.23 |
| ELK1_02 | 0.18 |
| ER_Q6 | 0.16 |
| FOXD3_01 | 0.12 |
| FOXJ2_02 | 0.17 |
| FREAC2_01 | 0.21 |
| FREAC3_01 | 0.19 |
| FREAC4_01 | 0.17 |
| FREAC7_01 | 0.15 |
| GATA2_01 | 0.26 |
| GATA3_01 | 0.22 |
| GATA_C | 0.23 |
| GRE_C | 0.13 |
| HFH3_01 | 0.12 |
| HLF_01 | 0.38 |
| HNF1_01 | 0.22 |
| HNF4_01 | 0.10 |
| HSF1_01 | 0.25 |
| HSF2_01 | 0.27 |
| IRF1_01 | 0.12 |
| ISRE_01 | 0.09 |
| LMO2COM_02 | 0.40 |
| MAX_01 | 0.24 |
| MEF2_03 | 0.18 |
| MEF2_04 | 0.12 |
| MEIS1_01 | 0.12 |
| MIF1_01 | 0.17 |
| MYB_Q6 | 0.24 |
| MYCMAX_01 | 0.19 |
| MYOD_Q6 | 0.09 |
| MZF1_01 | 0.08 |
| NF1_Q6 | 0.10 |
| NFAT_Q6 | 0.09 |
| NFE2_01 | 0.15 |
| NFKAPPAB_01 | 0.11 |
| NFY_01 | 0.25 |
| NKX61_01 | 0.15 |
| NRSF_01 | 0.04 |
| OCT_C | 0.16 |
| P300_01 | 0.11 |
| P53_01 | 0.09 |
| PAX2_01 | 0.29 |
| PAX5_01 | 0.05 |
| PAX6_01 | 0.28 |
| PBX1_02 | 0.24 |
| RFX1_01 | 0.22 |
| RORA1_01 | 0.18 |
| RORA2_01 | 0.20 |
| RREB1_01 | 0.04 |
| SOX9_B1 | 0.20 |
| SP1_Q6 | 0.04 |
| SREBP1_02 | 0.13 |
| SRF_Q6 | 0.17 |
| SRY_02 | 0.17 |
| STAT_01 | 0.17 |
| TATA_01 | 0.28 |
| TCF11_01 | 0.20 |
| TST1_01 | 0.24 |
| USF_01 | 0.19 |
| XBP1_01 | 0.48 |
| YY1_02 | 0.10 |

Approximate overestimation rates of position weight matrices from Patser
